# Giant primary retroperitoneal teratoma in an adult female patient: A case report

**DOI:** 10.3892/ol.2013.1374

**Published:** 2013-06-04

**Authors:** XIAO HUANG, BEN LIU, LIPING XIE

**Affiliations:** Department of Urology, The First Affiliated Hospital, College of Medicine, Zhejiang University, Hangzhou, Zhejiang 310003, P.R. China

**Keywords:** retroperitoneal teratoma, primary, adult

## Abstract

The retroperitoneum is an uncommon location for teratoma in adults. The current study presents the case of a rare giant primary retroperitoneal teratoma in a 55-year-old female. The clinical manifestations, diagnosis and surgical treatment procedure of this case are retrospectively reviewed. The patient presented with a complaint of an abdominal palpable mass and fullness for 1 month. The patient suffered a massive hemorrhage during the first exploratory laparotomyand only a small section of the tumor was resected. Pathology revealed a mature retroperitoneal teratoma. Eleven months after the first surgery, the tumor was resected successfully at the second laparotomy. The surgical specimen was a large tumor mass measuring 22×18×10 cm in size and weighing 6 kg. At follow-up, the patient was in a stable condition. This case study highlights the importance of imaging for the development of a pre-operative strategy and performing a safe surgical excision in giant retroperitoneal teratoma cases.

## Introduction

Teratomas are congenital tumors consisting of derivatives from the ectoderm, endoderm and mesoderm germ cell layers (1,2). A teratoma is considered to be a non-seminomatous germ cell tumor and is typically located in either the sacrococcygeal region or in the gonads. Malignant mature cystic teratomas (0.2–2% of cases) have the potential to metastasize to sites such as the retroperitoneal lymph nodes and lung parenchyma (2,3). Retroperitoneal teratomas are commonly identified in early childhood, but are rarely reported in adults (1,2). Giant retroperitoneal teratomas in adults are even rarer, with only a few cases previously described in the literature. The current study presents the case of a giant retroperitoneal teratoma in a 55-year-old female. The teratoma was considered unresectable at the first exploratory laparotomy and was finally treated successfully with surgical resection in the second surgery. A review of the current clinical literature on this topic supports our management of this case. Written informed consent was obtained from the patient.

## Case report

### Clinical presentation and diagnosis

A 55-year-old female (gravida 4, para 3) was referred to the First Affiliated Hospital (Hangzhou, China) with a 11-month history of an abdominal palpable mass and no previous history of routine health examinations. The patient was diagnosed with a right retroperitoneal tumor and severe hydronephrosis, and consequently underwent an exploratory laparotomy for the purpose of excision at Yiwu Central Hospital on August 10, 2006. The tumor was not completely removed due to a severe hemorrhage during the surgery. However, a small section of the tumor was resected and a nephrostomy was performed for the hydronephrosis. The pathology report resulted in a diagnosis of a mature retroperitoneal teratoma. At 11 months after the first surgery, the patient was admitted to The First Affiliated Hospital, College of Medicine, Zhejiang University (Hangzhou, Zhejiang, China) for a whole tumor resection.

**Figure 1.**
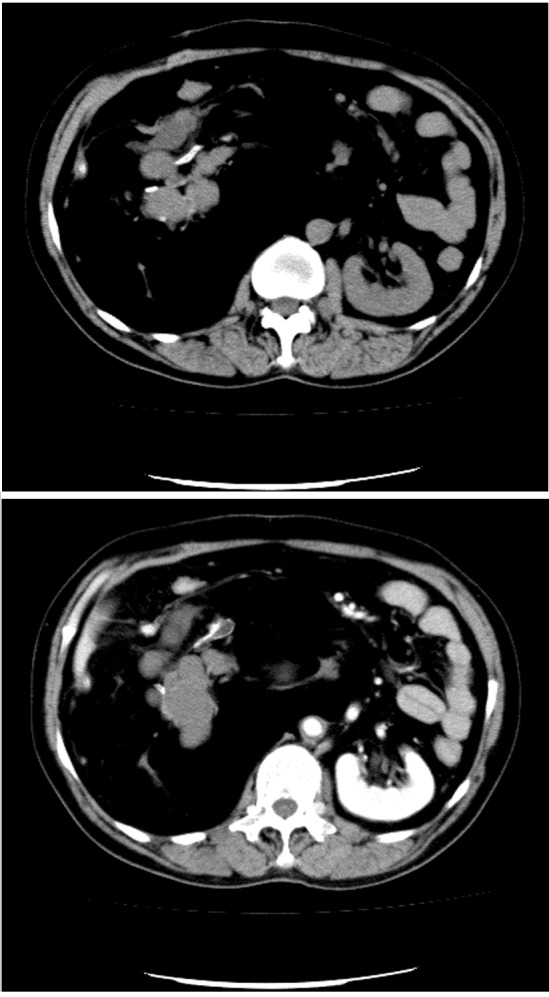
Computed tomography scans through the level of the celiac trunk reveals a giant retroperitoneal complex mass that was well-demarcated and contained a cystic room with fat, bone and tooth-like calcifications. The right kidney was encased by the tumor.

**Figure 2.**
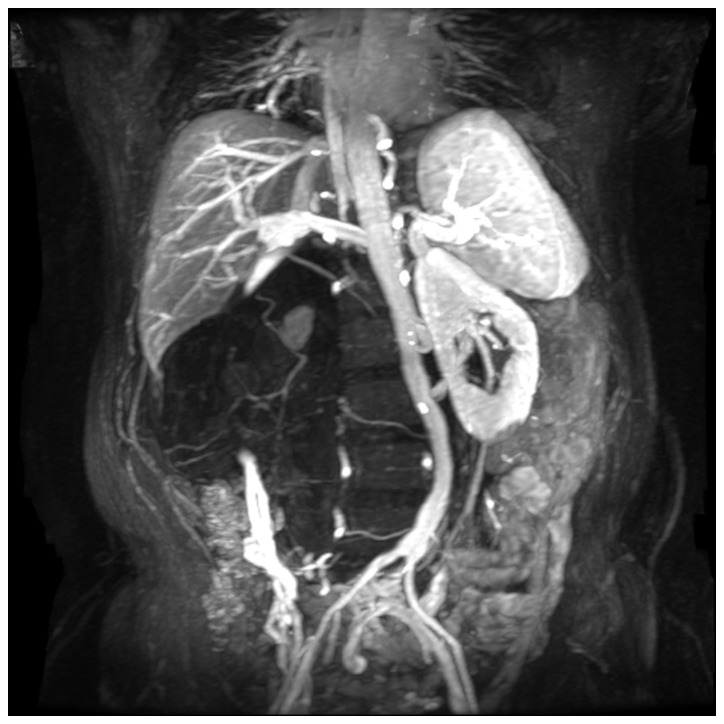
Magnetic resonance imaging (MRI) revealing the location and morphology of the tumor and the adjacent structures. The abdominal aorta was crushed to left and the inferior vena cava was narrowed. The ascending lumbar, azygos and hemiazygos veins were distended and twisted markedly.

A physical examination revealed a 25-cm mobile and solid mass with regular contours in the abdomen. A 20-cm vertical incision scar and a nephrostomy catheter were present on the right upper abdomen. The catheter drained ∼200 ml clear urine/day. Routine laboratory tests, including those for serum α-fetoprotein (AFP), were all within normal ranges. Abdominal ultrasonography revealed a parenchymatous irregular hypoechoic tumor in the right retroperitoneum. Abdominal spiral computed tomography demonstrated a large complex mass composed of multiloculated cystic components, soft tissue elements, inhomogeneous fatty tissue and calcifications in the right upper retroperitoneum. The remnant right kidney was encased by the tumor (Fig. 1). Magnetic resonance imaging (MRI) of the pelvis revealed a large 25×18×10-cm non-enhanced cystic mass in the retroperitoneum, distorting the abdomen severely at the level of the umbilicus and lifting the liver vessels from behind. The abdominal aorta was crushed to the left and the inferior vena cava was narrowed. The ascending lumbar, azygos and hemi-azygos veins were distended and twisted markedly (Fig. 2).

### Treatment and clinical course

**Figure 3.**
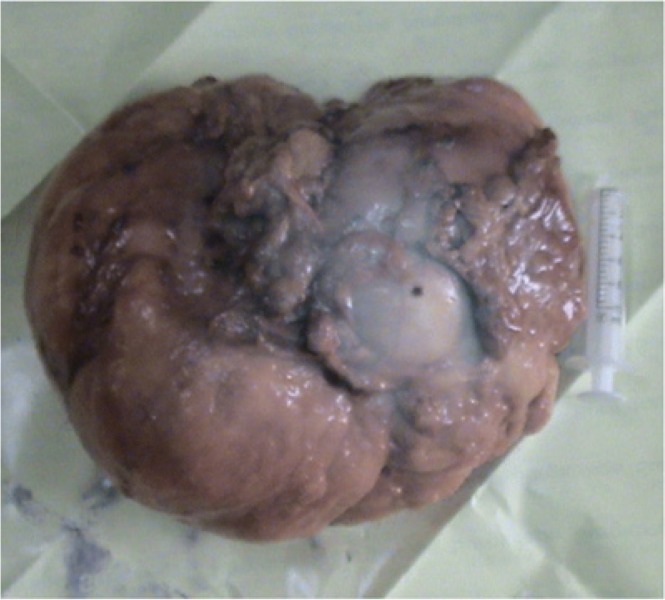
Appearance of the 22×18×10-cm tumor specimen.

The patient underwent a second laparotomy for the purpose of resection of the tumor on July 11, 2007. During the surgical procedure, a 25-cm long subcostal incision was made and the mass was found located in the retroperitoneal cavity, with adhesion to the adjacent structures. The tumor was resected successfully with a right nephrectomy. During surgery, the diaphragmatic muscle ruptured due to the adhesion of the mass, and thoracic closed drainage was performed. The patient had an uneventful post-operative recovery and was well at follow-up. The surgical specimen was a large tumor mass measuring 22×18×10 cm in size and weighing 6 kg. The specimen contained a hydronephrotic and distended kidney. The tumor consisted of fat, fiber and cartilage-like tissue (Fig. 3). Histological sections revealed mature, differentiated elements consistent with a mature teratoma. The kidney exhibited chronic pyelonephritis and atrophy, and pyelectasis was observed in the majority of the renal parenchyma.

## Discussion

Teratomas have been identified in individuals from every age group, however, the peak incidence is reported at between 20 and 40 years old (3). The majority of cases of malignant transformation are detected at 30–70 years old (4,5). Teratomas are generally divided into 2 histological types, mature and immature. A mature teratoma is an adult-type tumor consisting of differentiated elements, while an immature teratoma consists of elements with only partial somatic differentiation, similar to that observed in an embryo or fetus. In cases of retroperitoneal teratomas, ∼75% are benign and 25% are malignant. With regard to the histological subtypes, mature teratomas are generally benign, but may undergo malignant transformation into non-germ cell malignancies, including sarcomas and carcinomas (2,6). Immature teratomas have inherent malignant potential, but which percentage of immature teratomas in the retroperitoneum behaves in a malignant fashion is unclear.

The diagnosis of a retroperitoneal teratoma is often made on the basis of investigative imaging (2,7,8). Retroperitoneal teratomas are predominantly cystic or completely solid in appearance. Ultrasonography represents an important tool for making an early diagnosis and performing post-operative monitoring. Computed tomography (CT) scans or MRI are used to identify various components of these neoplasms, including soft-tissue density structures, adipose tissue and sebaceous and serous-type fluids. These imaging techniques are also able to indicate the precise location, morphology and adjacent structures of the tumor, enabling improved pre-operative planning and a more complete removal of the tumor with less damage (7). In addition, MRI, compared with CT, is more suitable for the determination of the association between the teratoma and celiac great vessels and the degree of tumor infiltration (8). AFP is produced by malignant retroperitoneal teratomas and functions as a specific tumor marker for laboratory diagnosis (6). Abnormal elevations in serum levels of carcinoembryonic antigen (CEA) and carbohydrate antigen (CA)19-9 have been reported in primary retroperitoneal teratomas (9). In the present case, all tumor markers were within the normal range.

The primary treatment of retroperitoneal teratomas is surgical resection (6,10). The most important structures in the abdomen are the aorta, vena cava, superior mesenteric vessels, celiac trunk and duodenum. Damage to these structures may cause overwhelming hemorrhaging, severe post-operative complications and even fatalities. Imaging of the tumor is critical for developing an effective pre-operative strategy and performing a safe surgical excision (6,9,10). As observed in the present patient, the benign teratoma expands and presses against, rather than encases, the surrounding structures and therefore may be dissected away from these adjacent structures. As with other abdominal surgeries, pre-operative imaging and the development of an appropriate strategy are essential for the excision of a retroperitoneal tumor. A malignant teratoma that invades the adjacent structures requires more extensive resection and may include the major vessels or organs. Unresectable or marginally resectable retroperitoneal teratomas may be shrunk following an initial course of chemotherapy (10).
